# Cyclicity of Binary Group Codes

**DOI:** 10.3390/e28030289

**Published:** 2026-03-04

**Authors:** Beatriz García García, Consuelo Martínez López, Ignacio F. Rúa

**Affiliations:** Department of Mathematics, University of Oviedo, 33007 Oviedo, Spain

**Keywords:** codes, rings, modules

## Abstract

In this paper, we study the cyclicity of binary group codes, identifying them as ideals in a group algebra. We focus on the construction of $\omega |\bar{\omega }$ codes, proving that they are self-dual group codes over the abelian group $C_{2}\times C_{k}$. We demonstrate that for even integers k>2, if the polynomial $x^{k}-1$ splits into self-reciprocal irreducible factors, these codes are not permutationally equivalent to any cyclic code. Additionally, we present computational results for binary group codes of length n<24 using the MAGMA software (V2.29-4). These results confirm that while all cyclic codes in this range are equivalent to abelian group codes, there exist non-cyclic group codes that cannot be realized as ideals in a cyclic group algebra, highlighting the strictly larger scope of the class of group codes.

## 1. Introduction

Error-correcting codes have gained importance in post-quantum cryptography, given that some promising post-quantum schemes are based on families of error-correcting codes with good properties. The first proposal in this direction, which has not yet been improved upon, uses binary Goppa codes. This is the well-known McEliece proposal [1].

Group codes are an important family that has been widely studied. In general, “group code” is used as a synonym for “left group code”; that is, the code can be identified with a left ideal of the group algebra F[G], where F is a finite field and *G* is a group. We have studied group codes, specifically, two-sided ideals of the group algebra F[G] in our research group. In particular, we have proved that there are group codes that can be obtained using a non-abelian group but cannot be obtained using abelian groups of the same order as *G* (see [2,3]). Furthermore, such codes exist over arbitrary finite fields (see [4]), and some of them have better parameters than any linear code with the same length and dimension. Specific decoding algorithms for group codes in the semisimple case have been studied (see [5,6,7]).

To better understand group codes, the MAGMA software [8] has been used to find all group codes over the field of two elements, $F_{2}$, up to permutation equivalence. We do this for the case of groups of length ≤23, given that we know that if |G|<24, every *G*-code can be viewed as an abelian group code, which facilitates calculations. A list of such group codes can be found in [9]. Therefore, we know the parameters (length, dimension, minimum distance, weight distribution, and a generator matrix) of all group codes of length <24.

## 2. Some Elementary Results

### 2.1. Group Codes

Hereinafter, F will denote a finite field and $G:=\{g_{1}=e,g_{2},\ldots ,g_{n}\}$ a finite group of order *n*. The set of all formal linear combinations of elements of *G*, $F\left[G\right]:=\left\{\sum_{g\in G}\alpha_{g}g\;\;:\;\;\alpha_{g}\in F\right\}$, has the structure of an algebra over F, called the *group algebra of G over *F. Its product is defined such that, given two elements $a=\sum_{g\in G}a_{g}g\in F\left[G\right]$ and $b=\sum_{g\in G}b_{g}g\in F\left[G\right]$:

$$a\cdot b=\sum_{g\in G}a_{g}g\cdot \sum_{h\in G}b_{h}h=\sum_{g\in G}\sum_{h\in G}\left(a_{g}b_{h}\right)\left(gh\right).$$

**Definition** **1.** *A linear code C over F is said to be a G-code of length n=|G| if there exists a bijective map ν:{1,…,n}→G mapping $i\to g_{i}$ such that $\left\{\sum_{i=1}^{n}a_{i}g_{i}:\left(a_{1},\ldots ,a_{n}\right)\in C\right\}$ is a two-sided ideal of F[G].*

**Definition** **2.** *A code C is called a group code if there exists a group G such that C is a G-code.*

**Remark** **1.** *If the ideal of F[G] is a right ideal (resp. a left ideal), the group code C it defines is said to be a right group code (resp. left group code). Thus, it will be understood that all ideals are two-sided unless specified as right or left.*

**Definition** **3.** *The group code C is called an abelian (resp. metabelian, cyclic, metacyclic, etc.) group code if there exists an abelian (resp. metabelian, cyclic, metacyclic, etc.) group G such that C is a G-code.*

Hereinafter, *n* will always denote the length of a code, which, in the case of a group code, will coincide with the order of the group; *k* will always denote the dimension of the code; and *d* its minimum distance. The weight distribution of a code will be denoted by WD and will be the set of pairs 〈w,s〉, where *s* is the number of codewords of weight *w*. Furthermore, an [*n*,*k*,*d*]-code denotes a code of length *n*, dimension *k*, and minimum distance *d*.

### 2.2. The Dual of a Group Code

Recall that if C is an [*n*,*k*]-linear code over the field F, the *dual code* of C is the [*n*,n−k]-linear code $C^{⊥}:=\left\{x\in F^{n}\;:\;x\cdot y=0\;\forall y\in C\right\}$, where · is the usual scalar product. It is said to be *self-dual* if $C^{⊥}=C$. Furthermore, if *M* is a generator matrix of the code C, then the transpose of *M*, $M^{T}$, is a parity-check matrix of its dual code, and $C^{⊥}:=\left\{x\in F^{n}\;:\;xM^{T}=0\right\}$ (see [10], pp. 1–37). A characterization of linear codes that are group codes can be found in [11]:

**Proposition** **1.** *Let C be a linear code of length n over a field F and G a finite group of order n.*

*1*. *C is a left G-code if and only if G is isomorphic to a transitive subgroup of $S_{n}$ contained in PAut(C).*
*2*. *C is a G-code if and only if G is isomorphic to a transitive subgroup H of $S_{n}$ such that $H\cup C_{S_{n}}\left(H\right)\subseteq \mathrm{PAut}\left(C\right)$, where $C_{S_{n}}\left(H\right)$ denotes the centralizer of H in $S_{n}$.*

**Corollary** **1.** *Since C and $C^{⊥}$ have the same permutation automorphism group, C is a G-code if and only if $C^{⊥}$ is also a G-code, where G is a group of order n.*

It is clear that for any length *n*, there exists only one group code of dimension 1: the repetition code [n,1,n], which is cyclic and associated with the ideal $\langle g_{1}+\cdots +g_{n}\rangle$ of F[G], $G=\{g_{1}=e,g_{2},\ldots ,g_{n}\}$. Likewise, there exists only one group code of dimension n−1, corresponding to the parity-check code [n,n−1,2]. Note that the latter is the dual of the repetition code. Thus, the study of group codes can be restricted to dimensions *k* between 2 and n−2, and taking duality into account, it is possible to limit the analysis to dimensions $k\leq \lfloor \frac{n}{2}\rfloor$.

### 2.3. Cyclic and Non-Cyclic Group Codes

The notion of a group code naturally extends that of a cyclic code. Recall that a linear code C is *cyclic* if it satisfies that $(a_{0},a_{1},\ldots ,a_{n-1})\in C$ if and only if $(a_{1},\ldots ,a_{n-1},a_{0})\in C$. Thus, a linear cyclic code is a *G*-code where *G* is a cyclic group. Another alternative view of these codes is through polynomial codes. In this way, a linear cyclic code can be seen as an ideal in the quotient ring $F\left[x\right]/\langle x^{n}-1\rangle$, generated by a (unique) monic polynomial g(x) such that $g\left(x\right)|\left(x^{n}-1\right)$ (see [10], pp. 188–215). The decomposition of this polynomial into irreducible factors allows for the generation of cyclic codes of a certain length and dimension.

In this study, we work with codes up to permutation equivalence. Thus, if $C_{1}$ and $C_{2}$ are two linear codes over F of the same length *n*, we say that $C_{1}$ and $C_{2}$ are *permutationally equivalent,* denoted $C_{1}∼C_{2}$, if there exists a permutation σ of the set {1,…,n} such that $\left(a_{1},\ldots ,a_{n}\right)\in C_{1}$ if and only if $\left(a_{\sigma (1)},\ldots ,a_{\sigma (n)}\right)\in C_{2}$ (see [10], pp. 188–215). Similarly, if *M* is a generator matrix of $C_{1}$, then $C_{1}$ and $C_{2}$ are equivalent if and only if MP is a generator matrix of $C_{2}$, where *P* is a permutation matrix [12].

**Definition** **4.** Given a polynomial $g\left(x\right)=a_{m}x^{m}+a_{m-1}x^{m-1}+\ldots +a_{1}x^{1}+a_{0}$, its reciprocal polynomial, denoted $g^{*}\left(x\right)$, is calculated as: $g^{*}\left(x\right)=x^{m}g\left(\frac{1}{x}\right)=a_{0}x^{m}+a_{1}x^{m-1}+\ldots +a_{m-1}x^{1}+a_{m}$. It is said to be self-reciprocal if $g\left(x\right)=g^{*}\left(x\right)$.

**Proposition** **2.** *Two cyclic linear codes generated by reciprocal polynomials are permutationally equivalent.*

**Proof.**  Let $C_{1}$ be an [n,k]-cyclic code over F generated by $g\left(x\right)=\sum_{i=0}^{m}a_{i}x^{i}$, where m=n−k. By definition (see [10], pp. 188–215), the code $C_{2}$ generated by the reciprocal polynomial $g^{*}\left(x\right)=\sum_{i=0}^{m}a_{m-i}x^{i}$ is the reciprocal code of $C_{1}$. This implies that a codeword $\left(c_{0},c_{1},\ldots ,c_{n-1}\right)$ belongs to $C_{1}$ if and only if the codeword $\left(c_{n-1},c_{n-2},\ldots ,c_{0}\right)$ belongs to $C_{2}$. Since the operation of coordinate reversal is a permutation of the component indices (specifically the permutation σ(i)=n−1−i for i=0,…,n−1), $C_{1}$ and $C_{2}$ are permutationally equivalent. □

## 3. Some Code Constructions

### 3.1. The Product Code

Recall that the *Kronecker product* of two matrices, $A=\left(a_{ij}\right)\in M_{p\times q}\left(F\right)$ and $B=\left(b_{ij}\right)\in M_{r\times s}\left(F\right)$, denoted by A⊗B, is performed by replacing each element $a_{ij}$ of matrix *A* with the block formed by the product of that element $a_{ij}$ and the entire matrix *B*. This results in a matrix of size equal to the product of the matrix sizes, $A⊗B\in M_{pr\times qs}\left(F\right)$. In this way, the *product code* of two linear codes, $C_{1}⊗C_{2}$, is constructed. If $C_{1}$ is an $\left[n_{1},k_{1}\right]$-linear code with generator matrix $M_{1}\in M_{k_{1}\times n_{1}}\left(F\right)$ and $C_{2}$ is an $\left[n_{2},k_{2}\right]$-linear code with generator matrix $M_{2}\in M_{k_{2}\times n_{2}}\left(F\right)$, the product code admits $M_{1}⊗M_{2}\in M_{k_{1}k_{2}\times n_{1}n_{2}}\left(F\right)$ as a generator matrix. It will have length equal to the product of the lengths and dimension equal to the product of the dimensions. Furthermore, this product of codes is associative but not commutative, although the product code $C_{1}⊗C_{2}$ is permutationally equivalent to $C_{2}⊗C_{1}$ (see [10], pp. 567–580).

### 3.2. $\omega |\bar{\omega }$ Codes

**Definition** **5.** *Let k be a positive integer. The $\omega |\bar{\omega }$ code is the binary linear code C of length 2k and dimension k that admits as a generator matrix the matrix whose first k columns form the identity matrix and the last k columns form the matrix with zeros on the diagonal and ones on the off-diagonal elements. That is, the $\omega |\bar{\omega }$ code admits the matrix $M:=(I_{k}|\bar{I_{k}})$ as a generator matrix, where:*

$$\bar{I_{k}}=\left(\begin{array}{ccccc} 0 & 1 & \ldots & 1 & 1\\ 1 & 0 & \ldots & 1 & 1\\ \vdots & \vdots & \ddots & \vdots & \vdots \\ 1 & 1 & \ldots & 0 & 1\\ 1 & 1 & \ldots & 1 & 0 \end{array}\right)\in M_{k\;\times \;k}\left(F_{2}\right)$$

**Proposition** **3.** *If k is even, then the code $C=\omega |\bar{\omega }$ of length 2k and dimension k is an $F_{2}G$ group code, where G is the non-cyclic abelian group $C_{2}\times C_{k}$. Furthermore, C has minimum distance *4 *if k>2.*

**Proof.**  Let C be the code of $F_{2}^{2k}$ generated by the rows of $\left(I_{k}|\bar{I_{k}}\right)$. Then the permutations $\sigma =\left(1,2,\ldots ,k\right)\left(k+1,\ldots ,2k\right)\in S_{2k}$ and $\tau =\left(1,k+1\right)\ldots \left(k,2k\right)\in S_{2k}$ have order *k* and 2, respectively, and generate an abelian group 〈τ,σ〉 isomorphic to $C_{2}\times C_{k}$, and $\langle \tau ,\sigma \rangle =C_{S_{2k}}\left(\langle \tau ,\sigma \rangle \right)\subseteq \mathrm{PAut}\left(C\right)$. By Proposition 1 the $\omega |\bar{\omega }$ code is an $F_{2}\left[C_{2}\times C_{k}\right]$ group code. Every word in C is the sum of elements in B, the basis linked to the matrix *M* (see Definition 5). If the word is the sum of *r* elements in B, then the weight of the word is 2r if *r* is even and *k* if *r* is odd. In particular, if k>2, every element in C has weight ≥4, and elements obtained with r=2 have weight 4. □

**Proposition** **4.** *If k is even, the code $C=\omega |\bar{\omega }$ is self-dual.*

**Proof.**  C is self-dual; it suffices to check that all rows of $M=(I_{k}|\bar{I_{k}})$ are orthogonal to each other. □

**Proposition** **5.** *Let k>2 be an even integer. If the polynomial $x^{k}-1\in F_{2}\left[x\right]$ only admits self-reciprocal irreducible factors, then the code $C=\omega |\bar{\omega }$ of dimension k is not permutationally equivalent to a cyclic code; that is, the code is not a cyclic group code.*

**Proof.**  Suppose, for the sake of contradiction, that C is permutationally equivalent to a cyclic code D. We know from Proposition 4 that C is a self-dual code. Since permutation equivalence preserves the dot product and dimension, the cyclic code D must necessarily be self-dual.

A cyclic code of length n=2k is self-dual if and only if its generator polynomial g(x) (which is a divisor of $x^{2k}-1$ of degree *k*) satisfies the self-duality condition:

$$g\left(x\right)g^{*}\left(x\right)\equiv 0\;\left(\mathrm{mod}\;x^{2k}-1\right)\Rightarrow g\left(x\right)g^{*}\left(x\right)=x^{2k}-1$$

where $g^{*}\left(x\right)$ denotes the reciprocal polynomial of g(x). In a field of characteristic 2, we have the identity $x^{2k}-1={\left(x^{k}-1\right)}^{2}$, so the condition is rewritten as:

(1) $$g\left(x\right)g^{*}\left(x\right)={\left(x^{k}-1\right)}^{2}$$

Let us analyze the structure of g(x) under the hypothesis of the statement. Let

$$x^{k}-1=\prod_{i}p_{i}{\left(x\right)}^{e_{i}}$$

be the decomposition into irreducible factors. By hypothesis, each factor $p_{i}\left(x\right)$ is self-reciprocal, that is, $p_{i}^{*}\left(x\right)=p_{i}\left(x\right)$. Since g(x) divides ${\left(x^{k}-1\right)}^{2}$, its irreducible factors must belong to the set $\left\{p_{i}\left(x\right)\right\}$. Since all these factors are self-reciprocal, the product of any combination of them will also be self-reciprocal. Consequently, g(x) is necessarily self-reciprocal:

$$g^{*}\left(x\right)=g\left(x\right)$$

Substituting this into Equation (1), we obtain:

$${\left(g\left(x\right)\right)}^{2}={\left(x^{k}-1\right)}^{2}$$

Taking the square root (a unique operation in characteristic 2, since the Frobenius endomorphism is injective), we conclude that the generator polynomial is unique:

$$g\left(x\right)=x^{k}-1$$

If the generator is $g\left(x\right)=x^{k}-1$, the code D contains the codeword corresponding to the polynomial:

$$c\left(x\right)=1\cdot g\left(x\right)=x^{k}-1\equiv x^{k}+1\;\left(\mathrm{mod}\;2\right)$$

The weight of this codeword is w(c)=2. This implies that the minimum distance of the cyclic code is d(D)≤2. However, by Proposition 3, we know that for k>2 (and even), the minimum distance of the original code is d(C)=4. This contradiction (2≥d(D)=d(C)=4) demonstrates that the initial assumption is false. Therefore, C cannot be equivalent to a cyclic code. □

**Corollary** **2.** *If k=2p, with p a prime number such that *2 is a primitive root modulo p (that is, $\langle 2\rangle =F_{p}^{*}$), then the code $C=\omega |\bar{\omega }$ of length 2k and dimension k is not permutationally equivalent to any cyclic code.

**Proof.**  Consider the factorization of $x^{k}-1$ over $F_{2}$. Given that k=2p, we have $x^{2p}-1={\left(x^{p}-1\right)}^{2}={\left(\left(x-1\right)\Phi_{p}\left(x\right)\right)}^{2}$. The condition $\langle 2\rangle =F_{p}^{*}$ guarantees that the cyclotomic polynomial $\Phi_{p}\left(x\right)=\sum_{i=0}^{p-1}x^{i}$ is irreducible in $F_{2}\left[x\right]$. The result follows from the previous proposition. □

## 4. Cyclicity of Binary Abelian Group Codes

As a particular case of computational interest, it has been verified that for any positive integer n<24, every cyclic linear code C of length *n* over the field of two elements $F_{2}$ is permutationally equivalent to a group code over the group algebra $F_{2}G$, for any non-cyclic abelian group *G* of order *n*. This verification has been carried out exhaustively using the MAGMA software [8]. For each length n<24 and each dimension $2\leq k\leq \lfloor \frac{n}{2}\rfloor$, all existing binary linear cyclic codes have been generated. Subsequently, it has been verified that each of them is, up to permutation equivalence, a group code over the algebra $F_{2}G$ for any non-cyclic abelian group *G* of order *n*. This allows us to ask whether this fact holds for non-cyclic binary group codes and whether it can be generalized to non-abelian groups or extended to other lengths.

In the case of non-cyclic abelian binary group codes, this fact does not hold. There exist non-cyclic group codes that cannot be viewed as *G*-codes for some abelian group *G*. For example, if $G=\langle a\rangle \times \langle b\rangle ≅C_{4}\times C_{4}$, in $F_{2}G$ we can consider the ideal generated by $1_{G}+b+b^{2}+b^{3}$ and $1_{G}+a+a^{2}+a^{3}$. The corresponding *G*-code C is a [16,7,4]-binary code, with weight distribution

WD={〈0,1〉,〈4,8〉,〈6,16〉,〈8,78〉,〈10,16〉,〈12,8〉,〈16,1〉}

and is non-cyclic.

Indeed, the only binary cyclic codes with parameters [16,7,4] are permutationally equivalent to the code generated by the polynomial $g\left(x\right)={\left(x+1\right)}^{9}=x^{9}+x^{8}+x+1$, whose weight distribution is

$$WD_{2}=\left\{\langle 0,1\rangle ,\langle 4,28\rangle ,\langle 8,70\rangle ,\langle 12,28\rangle ,\langle 16,1\rangle \right\}.$$

Therefore, C cannot be equivalent to a cyclic code. This code cannot be viewed as a *K*-code for the group $K=C_{2}\times C_{8}$, but it can be viewed as an *H*-code for $H=C_{2}\times C_{2}\times C_{4}$ or $H=C_{2}\times C_{2}\times C_{2}\times C_{2}$.

If $H=\langle a\rangle \times \langle b\rangle \times \langle c\rangle ≅C_{2}\times C_{2}\times C_{4}$, the code C is permutationally equivalent to the *H*-code defined by the ideal generated by $1_{H}+c+c^{2}+c^{3}$ and $1_{H}+a+b+ab$. And if $H=\langle a\rangle \times \langle b\rangle \times \langle c\rangle \times \langle d\rangle ≅C_{2}\times C_{2}\times C_{2}\times C_{2}$, the code C is permutationally equivalent to the *H*-code defined by the ideal generated by $1_{H}+a+d+ad$ and $1_{H}+b+c+bc$.

Similarly, if $G=C_{2}\times C_{2}\times C_{2}\times C_{2}$, the *G*-code defined by the ideal generated by $1_{G}+a+b+ba+c+d$ is a [16,8,4]-code with weight distribution

WD={〈0,1〉,〈4,12〉,〈6,64〉,〈8,102〉,〈10,64〉,〈12,12〉,〈16,1〉}.

This code is not permutationally equivalent to any other non-cyclic abelian code in $F_{2}\left[G\right]$ where *G* is an abelian group other than $G=C_{2}\times C_{2}\times C_{2}\times C_{2}$.

## 5. Group Codes Associated with a Non-Principal Ideal

In contrast to the semisimple case, in the non-semisimple case, there exist ideals that are not principal. In fact, there exist group codes that are not permutationally equivalent to any group code obtained via a principal ideal. For example, the [16,7,4]-binary group code C over $F_{2}\left[G\right]$ for $G=C_{4}\times C_{4}$ from the previous section is not permutationally equivalent to any group code linked to a principal ideal.

In other cases, there exist group codes constructed with a non-principal ideal that are permutationally equivalent to group codes obtained with principal ideals. For example, if $G=\langle a\rangle \times \langle b\rangle \times \langle c\rangle \times \langle d\rangle ≅C_{2}\times C_{2}\times C_{2}\times C_{2}$, in $F_{2}G$ we can consider the ideal generated by $1_{G}+a+b+c+ca+cb+d+dc$ and $1_{G}+a+b+ba+d+da+db+dba$.

The corresponding *G*-code D is a [16,5,8]-binary code, with weight distribution

WD={〈0,1〉,〈8,30〉,〈16,1〉}

and is non-cyclic. Indeed, the only binary cyclic codes have parameters [16,5,4] and are permutationally equivalent to the code generated by the polynomial $g\left(x\right)={\left(x+1\right)}^{11}=x^{11}+x^{10}+x^{9}+x^{8}+x^{3}+x^{2}+x+1$. Therefore, D cannot be equivalent to a cyclic code.

For this group code D, one cannot find a principal ideal of $F_{2}\left[G\right]$ with $G=C_{2}\times C_{2}\times C_{2}\times C_{2}$ that generates a code permutationally equivalent to it. However, one can find a principal ideal of $F_{2}\left[H\right]$ with $H=C_{2}\times C_{2}\times C_{4}$, $H=C_{4}\times C_{4}$, or $H=C_{2}\times C_{8}$, that generates a code permutationally equivalent to it.

If $H=\langle a\rangle \times \langle b\rangle \times \langle c\rangle ≅C_{2}\times C_{2}\times C_{4}$, the code D is permutationally equivalent to the *H*-code defined by the ideal generated by $c^{2}+a+b+ba+c^{3}+ca+cb+cba$. If $H=\langle a\rangle \times \langle b\rangle ≅C_{4}\times C_{4}$, the code D is permutationally equivalent to the *H*-code defined by the ideal generated by $1_{H}+a^{2}+a+ab^{2}+b+b^{3}+ba+ba^{3}$. And if $H=\langle a\rangle \times \langle b\rangle ≅C_{2}\times C_{8}$, the code D is permutationally equivalent to the *H*-code defined by the ideal generated by $b^{4}+b^{6}+a+ab^{2}+b+b^{3}+ba+b^{3}a$.

## 6. Some Properties

In [13], it is shown that every abelian group code associated with a minimal ideal in a semisimple algebra is permutationally equivalent to a cyclic one. The result is not true if the condition of minimality is removed. For example, if $G=\langle a\rangle \times \langle b\rangle ≅C_{3}\times C_{3}$, the ideal in $F_{2}\left[G\right]$ generated by $1_{G}+a+b+ab$ defines a [9,4,4]-code, which is the product code of the cyclic code in $C_{3}$ of length 3 and dimension 2 generated by the polynomial g(x)=x+1, with itself, and has weight distribution

WD={〈0,1〉,〈4,9〉,〈6,6〉}.

This code is not minimal (the only minimal ideals have dimensions 1 and 2) and is not cyclic.

What happens if we do not require the semisimplicity of the group algebra? For n<24 (where we know that all group codes are abelian group codes), the result remains true if $F=F_{2}$. However, it is not known what happens in the general case.

## 7. Conclusions

In this work, we have investigated the relationship between cyclicity and group codes over binary fields. We have formally defined and analyzed $\omega |\bar{\omega }$ codes, proving their self-duality and establishing conditions under which they are strictly non-cyclic. Our computational survey of codes with length n<24 confirms that while every binary cyclic code in this range is equivalent to an abelian group code, the class of group codes is strictly larger, containing optimal codes that do not possess a cyclic structure. These findings highlight the richness of group codes beyond the cyclic case. Future research will focus on extending these classifications to non-abelian groups and exploring the decoding capabilities of these non-cyclic structures.

## Data Availability

The data presented in this study are available on request from the corresponding author. The data are not publicly available due to privacy restrictions.
